# Does domestic violence during pregnancy influence the beginning of complementary feeding?

**DOI:** 10.1186/s12884-020-03144-y

**Published:** 2020-08-05

**Authors:** Gabriele Luiza Caprara, Juliana Rombaldi Bernardi, Vera Lúcia Bosa, Clécio Homrich da Silva, Marcelo Zubaran Goldani

**Affiliations:** Center for Child and Adolescent Health Studies – Hospital de Clínicas de Porto Alegre – Faculdade de Medicina, Universidade Federal do Rio Grande do Sul, Rua Ramiro Barcelos, 2350, Porto Alegre, RS 90035-903 Brazil

**Keywords:** Domestic violence, Pregnancy, Postpartum period, Complementary feeding

## Abstract

**Background:**

This study investigate the influence of domestic violence against pregnant women on early complementary feeding and associated factors.

**Methods:**

A longitudinal observational study was conducted with a convenience sample recruited from three public hospitals in Porto Alegre, Rio Grande do Sul, Brazil. Data on maternal age, education, marital status, breastfeeding, introduction of complementary feeding and domestic violence during pregnancy were investigated at four follow-ups points. Data on domestic violence was collected through a self-report questionnaire based on the Abuse Assessment Screen. The early introduction of complementary feeding, characterized as occurring before or at 3 months of life, was verified through a questionnaire prepared by the research group. Data analysis involved Student’s *t*-test, the chi-square test and Cox regression and was carried out in Statistical Package for the Social Sciences program. The significance level was set at 5%.

**Results:**

A total of 232 mother-infant pairs participated in the analyses, and 15.1% of the mothers reported suffering some form of violence. Domestic violence was directly associated with maternal education, marital status, and health status during pregnancy. Domestic violence was not associated with maternal age or breastfeeding at 3 months after delivery. In the univariate analysis, domestic violence during pregnancy was associated with early complementary feeding (RR = 1.74; CI: 1.01–2.98). This effect disappeared after the model was adjusted in multivariate analysis.

**Conclusions:**

There was no relationship between domestic violence during pregnancy and early complementary feeding.

## Background

Violence against women is acknowledged worldwide as a public health concern and is a major risk factor for the long-term physical and mental health of women [1, 2].

Domestic violence during pregnancy is associated with health problems for both the mother and the newborn and may result in miscarriage [2–5], intrauterine growth restriction, risk of prematurity, lower birth weight [2, 4, 5], higher stress biomarker levels in the child [6], and repercussions on the mother’s physical and emotional state [2–5], which impair the quality of motherhood and the mother’s ability to address the child’s needs, including feeding behavior [7].

Breastfeeding duration and practices may be influenced by many factors, including maternal age, education, stress, and depressive symptoms [7]. A number of studies have found that mothers who suffer domestic violence have lower breastfeeding intention and initiation [7], as well as earlier cessation of exclusive breastfeeding, than those who do not [7–10]. The prevalence of exclusive breastfeeding in children under 6 months is 41.6% worldwide [11]. In Brazil it is 41%, with an average duration of 1.8 months, according to the Brazilian Ministry of Health’s Second Breastfeeding Prevalence Survey in State Capitals and the Federal District [12].

World Health Organization and Brazilian Ministry of Health guidelines for infant feeding recommend exclusive breastfeeding during the first 6 months of life, followed by nutritionally adequate and safe complementary feeding and continued breastfeeding up to age two or older [13, 14]. Complementary feeding refers to all solid or liquid foods offered to the infant in addition to breast milk [14]. It is known that the introduction of food prior to 4 months is associated with lifelong adverse health outcomes, such as cardiovascular diseases, food allergies, type 1 and 2 diabetes mellitus [15–17], obesity, and an increased risk of overweight [7, 18, 19]. Infants only reach the renal and gastrointestinal maturity necessary to receive and metabolize foods other than breast milk after 6 months of age [20].

In a study of Indian women and their children up to 6 months of age, the authors reported that mothers exposed to some form of domestic violence were more likely to offer solid foods to infants, but that this practice did not differ significantly according to exposure or non-exposure to violence [21]. However, few studies have investigated the relationship between domestic violence against mothers and the introduction of complementary feeding to their infants.

Therefore, the purpose of this study was to investigate the influence of domestic violence against pregnant women on early complementary feeding and associated factors.

## Methods

### Sample and data

This longitudinal observational study, nested in a project called “Impact of variations of the perinatal environment on the health of newborns in the first 6 months of life” (IVAPSA) [22, 23] was approved by the Research Ethics Committees of Hospital de Clínicas de Porto Alegre (HCPA) and the Grupo Hospitalar Conceição (protocols 11–0097 and 11–027, respectively). The study involved minimum risk for the participants, in accordance with Brazilian Ministry of Health/National Health Council resolution 466/2012.

Between 2011 and 2016, a convenience sample of mother-infant pairs was recruited 24 to 48 h postpartum from three municipal public hospitals in Porto Alegre, Brazil (HCPA, Hospital Fêmina, and Hospital Nossa Senhora da Conceição). The mothers gave written informed consent prior to participation.

The intention-to-treat approach was used in the initial recruitment and sample selection, since participants were allocated into a control group and an adverse intrauterine group that was subdivided as follows: a) diabetes mellitus: puerperal women diagnosed with type 1, type 2 or gestational diabetes mellitus [24]; b) hypertensive disorders: puerperal women diagnosed with preeclampsia, chronic hypertension with superimposed preeclampsia, chronic or gestational hypertension [25]; c) smoking: women who smoked during gestation; and d) intrauterine growth restriction: women whose newborns were below the 5th percentile of the Alexander Growth Curve [26]. For the present study, however, the analyses were carried out without this subdivision, since domestic violence may have occurred prior to the presented outcomes.

Puerperal women with HIV, preterm newborns, congenital diseases or who required hospitalization were excluded. Mothers who, after personal or telephone contact, stated they had no further interest in participation, as well as participants who failed to answer the domestic violence questionnaire, were considered missing. Data collection was performed by trained interviewers who underwent periodic retraining.

### Variables

Data on maternal age, education, marital status, planned pregnancy, number of children, number of prenatal visits, breastfeeding and complementary feeding were collected through questionnaires prepared by the research group and administered in the third and sixth postpartum months at the mother’s home and the HCPA Clinical Research Center, respectively.

Domestic violence during pregnancy was assessed with a self-report questionnaire developed by specialists [3]. The questionnaire was based on the Abuse Assessment Screen, [27] which has been translated to Portuguese and validated [28]. It was applied in the first and the sixth months postpartum through interviews conducted at the HCPA Clinical Research Center. It involved four main questions assessing the type of violence suffered: physical (physical aggression or assault with weapons); psychological (verbal aggression); and sexual (forced sexual intercourse). If these questions were answered in the affirmative, the women were asked whether the violence took place during pregnancy or not. The participant was considered to have suffered domestic violence while pregnant if she responded in the affirmative to any type of violence.

In the present study, early complementary feeding was defined as the introduction of foods prior to or at 3 months of age, depending on the date scheduled for the interview. This was evaluated with a questionnaire applied at home in the third month postpartum. In this questionnaire, information on breastfeeding was also collected and divided into two categories: infants who were exclusively breastfed (infants who received only breast milk); and infants who were breastfed (infants who received breast milk concomitantly with another type of milk or infant formula).

In all analyses, maternal age was considered full years at the time of the interview and was calculated as the difference between the date of birth reported by the mother and the date of the interview. Maternal education was calculated as complete years of study at the time of the postpartum interview. The participant’s marital status was divided into two categories: living with a partner (married or living together) or living without a partner (single, separated, divorced, widowed or unmarried). Planned pregnancy was calculated as either yes or no. In all analyses, the number of children included all self-reported daughters and sons, including the newborn. The number of prenatal visits was considered the number of prenatal consultations registered in the mother’s Ministry of Health documents.

### Statistical analyses

The data were analyzed in SPSS (Statistical Package for Social Sciences) 18.0. Continuous variables were described as mean and standard deviation (±SD), while categorical variables were presented as absolute and relative frequencies. Student’s *t*-test was used to compare domestic violence with continuous variables (maternal age and maternal education), while the chi-square test was used for categorical variables (marital status and breastfeeding at 3 months). A Cox regression model was used to evaluate factors associated with early complementary feeding over time. The significance level was set at 5% (*p* < 0.05).

## Results

A sample of 232 mother-infant pairs was evaluated, because they had information on domestic violence during pregnancy. The mothers’ mean age was 27.4 ± 6.7 years, with 9.6 ± 2.7 years of education. Sample characteristics are shown in Table 1.

**Table 1:** Sample characterization– IVAPSA study. Porto Alegre (2011–2016)

Variables	
**Maternal age (years)**^**a**^	27.4 ± 6.7
**Maternal education (years)**^**a**^	9.6 ± 2.7
**Domestic violence during pregnancy**^**b**^	
No	197 (84.9)
Yes	35 (15.1)
Total	232
**Marital status**^**b**^	
With partner	194 (83.6)
Without partner	38 (16.4)
Total	232
**Breastfeeding at 3 months**^**b**^	
Exclusive breastfeeding	86 (37.1)
Breastfeeding	128 (55.2)
Total ^c^	214
**Planned pregnancy**^**b**^	
Yes	91 (39.2)
No	141 (60.8)
Total	232
Number of children*^a^	2.58 ± 1.6
**Number of prenatal visits**^**a**^	8.45 ± 3.2

^a^ Mean ± SD; ^b^ Absolute and relative frequency (%); ^c^ This variable was calculated as 214 participants, since 18 participants did not provide this information. *IVAPSA* Impact of perinatal environment variations on the health of the newborn in the first 6 months of life. * This variable includes the children evaluated in this study

Regarding domestic violence during pregnancy, 15.1% (35) of the mothers reported suffering some form of violence. The distribution of maternal characteristics in relation to domestic violence during pregnancy is described in Table 2. There was a statistically significant difference in maternal education and marital status (*p* = 0.044 and *p* = 0.018, respectively) between women who reported having suffered violence and those who did not.

**Table 2:** Distribution of maternal characteristics in relation to domestic violence during pregnancy –IVAPSA study. Porto Alegre (2011–2016)

Variables		Domestic Violence		
	Total	Yes	No	***p***-value^**a**^
**Maternal age (years)**^**b**^	27.4 (±6.7)	25.4 (±7.2)	27.7 (±6.6)	0.055
**Maternal education (years)**^**b**^	9.6(±2.7)	8.7 (±2.9)	9.7 (±2.6)	0.044
**Breastfeeding**^**c**^				
EBF	86 (40.2)	10 (32.3)	76 (41.5)	0.438
BF	128 (59.8)	*21 (67.7)*	107 (58.5)	
**Marital status**^**c**^				
With partner	194 (83.6)	24 (68.6)	170 (86.3)	0.018
Without partner	38 (16.4)	11 (13.7)	27 (31.4)	
**Early Introduction to Complementary Feeding**	77 (33.2)	17 (22.1)	60 (77.9)	–
**Number of children**^**b**^	2.58 (±1.6)	2.29 (±1.3)	2.60 (±1.6)	0.268
**Number of prenatal visits**^**b**^	8.45 (±3.2)	8.43 (±3.5)	8.44 (±3.1)	0.986
**Planned pregnancy**^**c**^				
Yes	91 (39.2)	11 (12.1)	80 (87.9)	0.402
No	141 (60.8)	24 (17)	117 (83)	

^a^ Student’s t-test and chi-squared test; ^b^ Mean ± SD, ^c^ Absolute and relative frequencies (%). *IVAPSA* Impact of perinatal environment variations on the health of the newborn in the first 6 months of life, *EBF* Exclusive Maternal Breastfeeding, *BF* Breastfeeding

A total of 33.2% (77) of the mothers reported having begun complementary feeding before the infant completed 3 months of life (mean days until onset 72.86 ± 17.83), of whom 22.1% (17) reported suffering some type of violence during pregnancy. Of all mothers who reported domestic violence during pregnancy, 51.4% (18) did not initiate early feeding.

Table 3 presents the univariate and multivariate analyses for early complementary feeding through Cox regression analysis. The dependent variable was the time in days at which each child began complementary feeding. In the univariate analysis, the number of children and the number of prenatal consultations were not statistically significant. The significant variables, including reported domestic violence, were included in the multivariate analysis. Following the adjusted analysis, when the combined effect of the variables was considered, domestic violence, planned pregnancy and marital status lost statistical significance (*p* = 0.535, *p* = 0.054 and *p* = 0.614, respectively). It was found that each year of maternal age and maternal education, as well as exclusive breastfeeding at 3 months of age were protective factors against early complementary feeding (HR = 0.963, HR = 0.890, HR = 0.469, respectively).

**Table 3:** Variables associated with early introduction of complementary feeding in infants – IVAPSA study. Porto Alegre (2011–2016)

Variables	Univariate Analysis*			Multivariate Analysis*		
	HR	95%CI	***p***-value ^**a**^	Adjusted HR	Adjusted 95% CI	Adjusted ***p***-value ^**a**^
**Maternal age**	0.94	[0.91; 0.98]	0.001	0.96	[0.93; 1.00]	0.039
**Maternal education**	0.86	[0.81; 0.94]	< 0.001	0.89	[0.81; 0.97]	0.009
**Breastfeeding**	2.34	[1.38; 3.98]	0.002	0.46	[0.27; 0.80]	0.005
**Marital status**	1.72	[1.03; 2.90]	0.039	1.16	[0.65; 2.06]	0.614
**Domestic violence**	1.74	[1.01; 2.98]	0.044	1.21	[0.66; 2.23]	0.535
**Number of children**	1.04	[0.91;1.19]	0.531	1.03	[0.90;1.17]	0.649
**Number of PN visits**	0.97	[0.91;1.04]	0.490	0.99	[0.92;1.05]	0.763
**Planned pregnancy**	0.62	[0.38;0.99]	0.045	0.62	[0.39;1,00]	0.054

^a^ Cox regression. *IVAPSA* Impact of perinatal environment variations on the health of the newborn in the first 6 months of life, *HR* Risk ratio, *95%CI* 95% confidence interval, *PN* prenatal. * All variables included in the univariate analysis were included in the multivariate analysis. The dependent variable was the time in days at which each child began complimentary feeding

## Discussion

In the present study, no significant association was found between early complementary feeding and domestic violence during pregnancy. Most participants who began early complementary feeding reported no violence during pregnancy. The prevalence of early complementary feeding was 33.2%, which was higher than the 7% found by Magarey et al. [29]. This difference could be explained by the fact that our sample of mothers were younger and had less education. This is in line with previous publications that have found an association between low education, lower maternal age, and early complementary feeding [14, 15, 30–32].

Domestic violence, particularly from an intimate partner, has been studied as a risk factor for inadequate breastfeeding practices [7–10], and such practices are related to the introduction of complementary feeding. We found that, following a multivariate analysis, exclusive breastfeeding at 90 days was a protective factor against early complementary feeding. Thus, there is a greater risk of early complementary feeding in infants who are not exclusively breastfed [14, 30, 33]. There was no significant association between exclusive breastfeeding and domestic violence during pregnancy.

According to previous studies, maternal education is associated with domestic violence in pregnancy. Lower levels of maternal education have been associated with a higher prevalence of domestic violence during pregnancy [4, 5, 33]. This finding was confirmed in the present study: domestic violence during pregnancy was significantly more prevalent in women with a low education level. The number of previous children (i.e. whether or not the mother is primiparous) was not associated with domestic violence or early complementary feeding, nor were the number of prenatal visits, which could indicate whether the mother received professional guidance regarding postpartum care (including nutrition). Planned pregnancy was not associated with domestic violence or early complementary feeding (in the adjusted analysis), indicating that planning is not a protective or risk factor for domestic violence or early complementary feeding.

The relationship between marital status and domestic violence during pregnancy revealed that the presence of a partner was significantly associated with domestic violence, a result also found by Santos et al. [2]. Nevertheless, other authors have not found the same association, observing instead that exposure to violence was more prevalent in women who did not have a partner [10, 34]. There was no also significant association between complementary feeding and marital status after the adjusted analysis.

We found that each year of maternal education becomes a protective factor for the early introduction of complementary feeding. In fact, a number of studies have found low education to be associated with early complementary feeding [15, 16, 29]. Considering its long-term importance for the infant’s health, maternal access to infant feeding information at an appropriate reading level is a clear necessity, as has been strongly demonstrated in the literature [7, 15–19]. Another protective factor for early complementary feeding was maternal age: the older the mother, the lower the probability of beginning complementary feeding before 3 months of age. Previous research on the determinants of infant feeding practices has shown an association between early complementary feeding and young mothers [15, 31]. Similar to our findings. Scott et al. found that maternal age continued to be associated with early complementary feeding, even after adjustment for possible confounding factors [31]. It can be suggested that the lower the mother’s age, the greater the probability that she is insecure about her ability to care for the child, since older mothers may have more experience or previous children, which would facilitate introducing complementary feeding at a more appropriate time.

The main limitations and weaknesses of this study involved the domestic violence data, since the mothers were the sole source of information, and underreporting may have occurred. Consequently, the small number of participants who reported suffering domestic violence during pregnancy decreased the intensity of the observed associations. Some women might not have felt safe about reporting domestic violence during pregnancy due to personal issues such as embarrassment, fear, and economic dependence, or to social issues such as family privacy, victim blaming, etc. [35]. In a recent study by Caleyachetty et al. [36], underreporting of domestic violence was also cited as a limitation, which also could have attenuate their findings [36]. On the other hand, our study’s strength is that it has explored, in an unprecedented fashion, the relationship between domestic violence during pregnancy and early complementary feeding. In the future, a mechanism for determining the impact of an environment of violence on infant feeding could be explored.

## Conclusion

Thus, there was no relationship between domestic violence against pregnant women and early complementary feeding, since violence was only a risk factor for the early complementary feeding in the univariate analysis, but was not significant when adjusted for other factors. However, these conclusions should be interpreted with caution, since the sample size of victims was small.

A previous study also found that exposure to violence did not significantly affect complimentary feeding prior to 6 months of age [21]. An association between domestic violence and complementary feeding has not been established in greater detail due to the lack of studies on this subject [37].

The present study highlights the importance of identifying domestic violence risk groups during prenatal care, as well as during postpartum and childcare consultations. Through the diligence of maternal and child health professionals, actions targeting the early detection of domestic violence during pregnancy could be developed, preventing future aggravations to maternal and child health and identifying mothers at risk of early weaning/complementary feeding.

Although domestic violence during pregnancy has been the subject of many studies, new research could clarify the interaction between domestic violence and the onset of complementary feeding.

## Data Availability

The dataset used and analysed during the current study are available from the corresponding author on reasonable request.

## Abbreviations

HCPA: Hospital de Clínicas de Porto Alegre
IVAPSA: Impact of perinatal environment variations on the health of the newborn in the first 6 months of life
SD: Standard Deviation
HR: Risk ratio
EBF: Exclusive Maternal Breastfeeding
BF: Breastfeeding
SPSS: Statistical Package for Social Sciences
HIV: Human Immunodeficiency virus
